# Factors Associated with COVID-19 Vaccine Refusal: A Community-Based Study in the Menoua Division in Cameroon

**DOI:** 10.3390/tropicalmed8090424

**Published:** 2023-08-24

**Authors:** Aimé Césaire Momo Tetsatsi, Astride Arolle Nguena, Andrillene Laure Deutou, Alaric Tamuedjoun Talom, Beatrice Talom Metchum, Armand Tsapi Tiotsia, Pierre Watcho, Vittorio Colizzi

**Affiliations:** 1 Faculty of Science and Technology, Evangelical University of Cameroon, Bandjoun P.O. Box 127, Cameroon; 2 Research Unit of Animal Physiology and Phytopharmacology, University of Dschang, Dschang P.O. Box 67, Cameroon; 3 Faculty of Health Sciences, The University of Bamenda, Bambili P.O. Box 39, Cameroon; 4 Department of Biology and Interdepartmental Centre for Comparative Medicine, University of Rome Tor Vergata, 00133 Rome, Italy

**Keywords:** determinants, COVID-19 vaccination, Menoua Division, Cameroon

## Abstract

COVID-19, which was named in March 2020 as a global pandemic by the WHO, remains a serious public health threat worldwide. Despite the adoption of vaccines as an effective strategy to counter this pandemic, the vaccination rate in Cameroon is far lower than that planned by the Cameroonian government and its partners. The main objective of this study was to determine the factors limiting COVID-19 vaccine acceptance in the Menoua Division in the West Region of Cameroon. A community-based cross-sectional and analytical study was conducted between March and April 2022 in the Menoua Division. A pre-tested questionnaire was filled out by willing participants of more than 18 years old, and data were further expressed in order to estimate the knowledge of participants on COVID-19, vaccine status, and the factors associated with vaccine refusal. A Pearson test was performed in order to identify the associated factors, with a *p*-value < 0.05 considered as significant. A total of 520 participants with a mean age of 33.27 ± 12.78 were included. Most had a secondary education level (56.15%), and trade and informal sectors (34.04%) were the main occupations. Knowledge on COVID-19 was average, and it was significantly associated (*p* < 0.05) with gender and education level. The vaccination rate was 10%, which was six times less than the national target. A lack of information, confidence, and medicinal plant use were all factors significantly associated with vaccine refusal. This pioneer community-based study in Cameroon identified a lack of knowledge, confidence, and medicinal plant use as the leading factors limiting COVID-19 vaccine acceptance in Cameroon. Health authorities should therefore strengthen sensitization in order to tackle the lack of information and the misinformation among the target groups.

## 1. Introduction

The world was hit in December 2019 by a severe acute respiratory syndrome (SARS), which began in Wuhan, China. The disease was rapidly named, and was further qualified as a global pandemic by the World Health Organization (WHO) in March 2020 [1]. Recent data suggest that over 6.5 million people, including around 1935 in Cameroon, have died from COVID-19 since 2019 [1]. The first case of COVID-19 was registered in Cameroon on the 6 March 2020, and the government adopted a nationwide strategic plan to address the situation [2,3]. The specific COVID-19 transmission mode led to the establishment of barrier measures, including lockdowns, social distancing, widespread stay-at-home orders, isolating suspected cases, quarantining confirmed cases, face masks, and health education on handwashing and environmental hygiene measures in order to control its propagation [4]. Although the strategy proved to be effective in limiting the spread in China and Europe, the effect was very low in Cameroon due to non-observance of these restrictive measures [5]. Based on these reasons, anti-COVID-19 vaccination could be viewed as the best option for African populations to prevent the effects of the virus. 

Drug development obeys well-established rules, and it requires a long period to follow adequate steps. COVID-19 vaccination broke the record as the most promptly and massively deployed public health intervention in the history of healthcare [6]. The WHO planned to achieve a vaccination rate of 70% by June 2022. For African countries, a two-step strategy was adopted by at least 10% of the population by September 2021 and 70% by June 2022 [6]. The vaccination campaign was launched in Cameroon on 12 April 2021 with the objective of achieving a 60% vaccination rate in the population by December 2022 [7]. A total of 840 vaccination centers were set up across the country, and four types of vaccines, including BBIBP-CorV (Sinopharm), ChAdOx1, nCoV-19 adenoviral (AZD1222; Oxford- Astra Zeneca), JNJ-78436735 (Ad26.COV2. S; Johnson and Johnson), and BNT162b2 mRNA (Pfizer Biontech), were mobilized through facilitation platforms, such as the COVID-19 Vaccines Global Access initiative [7,8]. On 13 February 2022, the Expanded Program of Immunization of the Cameroon Ministry of Public Health reported that only 2.9% of the general population, of which 5.87% were of the target population, were vaccinated [7]. This figure is consistent with several reports which indicated that during the early stage of vaccination, contrary to 66.53% and 74.47% of COVID-19 vaccine acceptance rates in Burkina Faso and Nigeria, respectively, only 15% of Cameroonians were likely to take the vaccine [4,9]. With a 6% vaccination rate in September 2022, the West Region was far lower than the targeted rate [7,10]. This situation stresses the need to identify and monitor the factors limiting COVID-19 vaccination. 

Vaccine hesitancy is defined as the reluctance or refusal to take the vaccine despite accessibility [11]. Even if some studies have reported misinformation, mistrust of vaccine safety and effectiveness, and religion as just some of the factors associated with COVID-19 hesitancy, vaccine hesitancy is a context-dependent phenomenon that should be examined and monitored regarding local reality [4,12,13]. Previous studies have reported that in Cameroon, the lack of confidence in approved vaccines as well as concerns about vaccine side effects were associated with vaccine hesitancy amongst health personnel [6,14]. Still, no community-based study, to the best of our knowledge, has investigated the reason for COVID-19 vaccine refusal in Cameroon in general. In order to address this question, this study investigated the determinants of COVID-19 vaccine hesitancy in the Menoua Division, West Cameroon.

## 2. Materials and Methods

### 2.1. Study Design and Period

A community-based cross-sectional and analytical study was carried out in six (06) subdivisions of the Menoua Division––namely, Dschang, Fokoué, Fongo-Tongo, Nkong-Ni, Penka-Michel, and Santchou. This study was conducted from March to April 2022. 

### 2.2. Study Population 

The study population consisted of all citizens living in the Menoua Division at the time of the study. 

### 2.3. Inclusion and Exclusion Criteria

Men and women aged 18 and above who were willing to participate in the survey were included in the study. Vulnerable participants were excluded as well as those with incoherent data or incompletely filled questionnaires.

### 2.4. Sample Size and Data Collection Tool

In order to estimate a representative sample size for this study, the following formula was used:

(1) $$n=\frac{Z\alpha^{2}p\left(1-p\right)}{d²}$$

where *n* represents the sample size, *p* represents the prevalence, *Zα* represents the 95% confidence interval, and d represents the accepted margin error of 5%, assuming a prevalence rate of 50%. The sample size of 380 participants was recorded, and it was increased by 10% of the potential reluctance rate in order to provide a minimum sample size of 460 participants. This sample was proportionally partitioned into six subdivisions regarding population size. A pre-tested questionnaire was used as a data collection tool to register the sociodemographic features of the participants as well as their knowledge about COVID-19, vaccination status, and associated factors.

### 2.5. Data Collection Procedure

We performed a questionnaire-based survey as described by Tendongfor et al. [15], but with some changes. Contrary to Tendongfor et al., we choose a community-based approach in order to ensure an equal chance to all of the population categories to be represented. Data collection was carried out at different markets of each subdivision. Two trained investigators were mobilized during popular market days and popular church days (Sundays) for data collection. The self-administered method was the formal strategy, but for the participants with a language or literacy barrier, investigators were allowed to fill in the response sheet after receiving the verbal consent of the participant.

### 2.6. Data Processing and Analysis

At the end of each collection day, the questionnaire was double-checked by the investigation team and the supervisor. Coherent and fully filled questionnaires were classified and data were tipped in Excel format using Microsoft Office Excel 2013. Data were then exported to SPSS 20.0 Software for statistical analysis. Pearson chi-square test was performed in order to determine the association between the variables. Logistic regression was used in order to test the association level of the dichotomous variables referring to the presence of COVID-19 in Cameroon, the availability of vaccine knowledge and the nearest vaccination center, and the price of vaccines. The test was considered significant if *p* < 0.05.

To classify the knowledge of participants, those who named the principal signs and symptoms of COVID-19, preventive measures, the nearest vaccination center, and the type of vaccines were said to have a good knowledge of COVID-19 transmission, prevention, and vaccination, respectively. Those who cited uncommon signs and symptoms and none of the vaccine types were qualified as average knowledge. Participants ignoring signs and symptoms, preventive measures, the nearest vaccination point, and the vaccines available were said to be null.

## 3. Results

### 3.1. Study Population 

A representative sample of 520 participants took part in the study, which was 13.04% (*n* = 60) higher than the calculated sample size. Proportional to their population size, Dschang (34%, *n* = 177), Penka-Michel (25%, *n* = 130), and Nkong-Ni (20%, *n* = 104) were the most represented subdivisions. A total of 60.58% of the participants were female and 45.19% were single (Table 1).

The mean age of the participants was 33.27 ± 12.78, ranging from 18 to 96 years. A total of 50% (*n* = 260) of the participants were less than 30 years old and 11.15% (*n* = 58) were above 50 years old (Table 1). Participants were mostly of secondary education level (56.15%, *n* = 292) and trade, education, and informal sectors were the most represented professions. Health personnel accounted for 4.04% (*n* = 21) of the sample (Table 1).

### 3.2. Knowledge of the Participants on COVID-19 Manifestations 

As indicated in Table 2, 65.58% of the participants had "good” and 23.65% had “average” knowledge of COVID-19 manifestations. Contrary to age, the knowledge of COVID-19 manifestations was significantly (*p* < 0.005) associated with gender and educational level, as female participants with a low educational level were less informed (Table 2).

### 3.3. Knowledge of the Participants about COVID-19 Prevention

The majority of the participants (72.31%, *n* = 376) had average knowledge regarding COVID-19 prevention. A total of 266 (34.54%) declared that they used medicinal plants and foods as a preventive method, and the most cited were ginger and lemon with 27.62% (*n* = 100) and 19.06% (*n* = 69), respectively (Table 3). 

### 3.4. Profile of Plant Users as a Preventive Method against COVID-19

Considering all of the participants (*n* = 520), 52.68% of the female participants used one or a combination of plant products compared to 50.16% of their male counterparts. Moreover, more than 55% of the participants above 30 years old used plant products, with a record of 56.90% for those aged 50 years plus. Considering the education level, proportions of 55.06% and 57.55% of plant users were recorded in groups of participants with primary and university education level, respectively (Table 4). Plant use was significantly associated with age. 

### 3.5. Knowledge and Attitude of the Participants to COVID-19 Vaccine 

Data from Table 5 indicate that 11.92% of the participants were not aware that vaccines were present in Cameroon, while 41.61% ignored the nearest vaccination point. Regarding the role of the vaccines, almost all of the participants (92.31%) declared that they prevented COVID-19 infection, but only 27.50% of the participants qualified the vaccines as being of good quality, and 26.35% indicated death as a major side effect of the vaccine, whilst cancer (4.04%) and sterility (6.15%) were also mentioned as other vaccine side effects. A large majority of the participants (58.12%) declared that they were worried about vaccine side effects (Table 5). 

### 3.6. Vaccinal Status of the Participants

**Figure 1:**
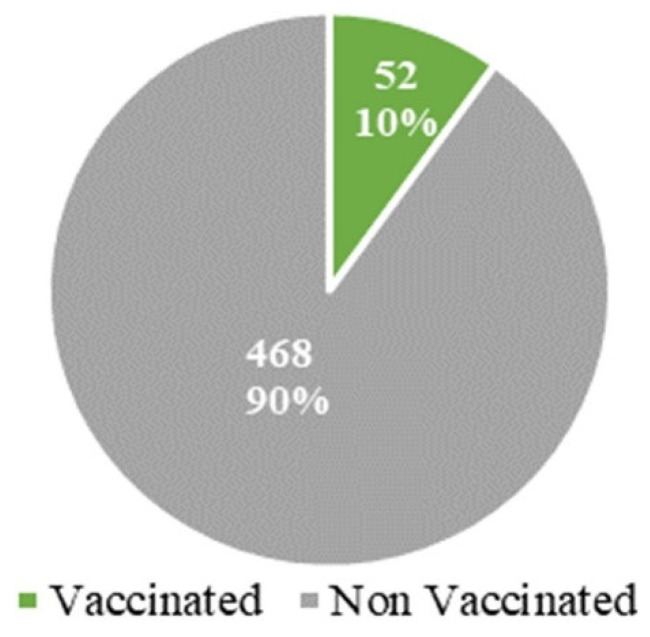
Vaccination status of participants.

Out of 520 participants in the study, only 52 were vaccinated, representing 10% of the total sample (Figure 1).

### 3.7. Profile of Vaccinated and Non-Vaccinated Participants

#### 3.7.1. Knowledge

Table 6 summarizes the knowledge of participants regarding the presence of COVID-19 in Cameroon, the availability of vaccines, vaccination centers, and vaccine cost. A total of 90 participants denied the presence of COVID-19 in Cameroon, with 93.34% of them not vaccinated. A strong and significant (*p* < 0.005) association was found between vaccine acceptation and knowledge of the availability of vaccines in Cameroon, the nearest vaccination point, and the cost of the vaccines. In fact, none of the participants who ignored vaccine accessibility and the cost were vaccinated. The logistic regression confirmed that participants who were not aware of a vaccination center were more than five times less likely to take the vaccines (odds ratio: 5.18).

#### 3.7.2. Attitude

Table 7 describes the association between the attitude of participants and vaccine acceptance. A statistically significant association (*p* < 0.005) was found between vaccine refusal and a participant’s belief regarding vaccine quality, side effects, and effective protection compared to medicinal plants. Almost all of the participants declared that the vaccines are of bad quality (97.97%) and that they have dangerous side effects (97.81%), and those with concerns about the side effects (96.17%) were not vaccinated. Furthermore, none of the participants using medicinal plants as a treatment option were vaccinated.

#### 3.7.3. Personal or Sociodemographic Factors

The expression of vaccine status per age group, gender, and education level showed that vaccine acceptance was significantly associated with both age and education level. A total of 93.08% of the participants aged less than 30 years old and 93.15% of secondary education level were not vaccinated. However, gender did not affect the decision to take the vaccine (Table 8).

## 4. Discussion

The present study evaluated factors associated with COVID-19 vaccine refusal in the Menoua Division from March to April 2022. A community-based cross-sectional and analytical survey was conducted, and a representative sample size of 520 participants were interviewed. Since the homologation of vaccines as the principal preventive approach to COVID-19, the WHO and governments around the world have faced many challenges to equitably distribute the vaccines [4]. Despite the effectiveness of many strategies that have been put in place to facilitate the distribution of the vaccines in Africa [7], an unpredicted resistance of the population to get vaccinated has been noticed [4,13]. As documented by other studies, many theories could explain this controversy, and factual evidence has pointed to poor information or misinformation as the major factors that spark fears of potential side effects of the vaccines [6,14,16].

Participants of the present study were mostly from Dschang, Penka-Michel, and Nkong-Ni due to the high demography of these subdivisions. Males represented 60.58% of the participants, and 54.81% of the participants were married; the latter merely reflects national statistics, where about 50% of people aged between 15 and 49 years old are married [17]. The study population was young, with a mean age of 33.27 ± 12.78, and with extremes of age from 18 to 96 years old. Only 11.15% of the participants were above 50 years old. The mean age of the participants was higher than the figure of the general population in Cameroon, and it was similar to the study population described by Aseneh et al. [14]. A secondary education level was the most prevalent in the sample, and traders as well as people from the educational and informal sectors were the dominant professions. The profile of our participants was similar to that of the population studied in Kenya by Shah et al. [18]. The high proportion of participants from the informal sector could be explained by the fact that the survey was conducted at the local market, while the frequency of teachers could reflect their readiness to participate in the survey due to their intellectual level. Indeed, Dinga et al. also reported teachers as the most represented category in their survey [16]. As a consequence of the collection site, health personnel accounted for only 4.04% of the sample.

As the awareness and accessibility of a product can highly influence the decision of patients or candidates [13], we evaluated the knowledge of participants regarding COVID-19 manifestations, prevention, and vaccination. Data revealed that participants had good knowledge of COVID-19 symptoms, and that this was significantly associated with gender, age, and education level. Despite the hesitancy of the population in respecting both barrier measures and the vaccination instructions to counter the pandemic, COVID-19 remains an important matter of concern across all social categories [5]. This awareness could be the result of several sensitization campaigns held by sanitary authorities, which were a result of the government’s response strategy against COVID-19 [7]. Contrary to COVID-19 manifestations, participants showed average knowledge (72.31%) regarding prevention of the virus, and many participants preferred medicinal plants, such as ginger, lemon, garlic, and artemisia as treatment options. The limited awareness of COVID-19 prevention is consistent with the limited respect that was shown towards the preventive methods observed nationwide [5,18,19]. For some, the pandemic has already passed, and for others, COVID-19 is not dangerous and/or does not exist in Cameroon [20].

Medicinal plants have long been used in Africa for health problems. This is not only sustained by cultural habits but also due to limited and affordable health services [12,20,21]. Contrary to gender and education level, plant use was significantly associated (*p* < 0.005) with age, as the preference for the use of plants increased with age. The effectiveness of medicinal plants has been scientifically discussed [21,22].

The evaluation of the knowledge/attitude of participants regarding vaccines revealed poor awareness of the participants of vaccination centers and the role of vaccines. They also claimed to be very worried about the side effects of the vaccines, which included, among others, cancer, sterility, and death. These data reflect the disinterest of the population towards vaccination, and they indicate how far conspiracy theories have found a fertile ground in Africa, where people are naturally reluctant to undergo some treatments [20]. Since the beginning of the pandemic, misinformation has been shared on social media that claims COVID-19 is a planned strategy nefariously designed to control the world population, especially in Africa [20,23]. This thesis gained massive attention when the WHO predicted on 17 April 2020 that Africa would become the next epicenter of the COVID-19 pandemic [24]. A similar refractory attitude to the one we have reported towards the COVID-19 vaccines was reported in Cameroon by Ajonina-Ekoti et al. before the vaccination campaign, where about 87% of the 591 participants reported their unwillingness to receive a COVID-19 vaccine if it was available in the country [25]. Almost all of the participants ignored the role of vaccines, as 92.31% said that the vaccine prevent contamination. Unlike other vaccines, COVID-19 vaccines do not protect against infection, but, rather, prevent against severe forms of the pathology [26]. Similarly, many people believe that medicinal plants have been proven to prevent severe forms and death from COVID-19 [21,22].

Data on the knowledge and attitude of populations regarding COVID-19 prevention and vaccination were a sign of the low vaccination rate in the population. Indeed, only 10% of our study population were vaccinated. Although higher than the data found in the West African regional and national statistics, this result is far lower than the mooted 70% by June 2022 and the 60% by December 2022 that were planned by the WHO and the Cameroonian government, respectively [8,10]. These results are consistent with the reports of Patwary et al. [9] and Ajonina-Ekoti et al. [25], which indicated that at the early stage of the vaccination campaign, contrary to the 66.53% and 74.47% of people that favored vaccination in Burkina Faso and Nigeria, respectively, only 15% of Cameroonians had declared that they were likely to take the COVID-19 vaccine. These data also corroborate the reports from Drescher et al. [20], which indicated that both Cameroonians and Ivorians expressed reluctance to vaccinate right before the vaccination campaigns began. Furthermore, Murewanhema et al. [27] suggested that ignorance sustains a limited acceptance of vaccines in Zimbabwe. In this study, participants were not questioned on the number of doses of the vaccine that were taken, and this information could have permitted us to differentiate participants that were partially vaccinated from those with full protection.

In respect to the objective of the present study, the vaccine status of participants was paired to some variables, and it appeared that the lack of information, the lack of safety or the lack of confidence regarding vaccines, as well as some sociodemographic features were all significantly associated with vaccine acceptance. These features have also been documented in numerous studies in Cameroon and Africa [13,16,21].

Lack of information as a major factor affecting vaccine refusal was evidenced in this study by a significant association (*p* < 0.005) between vaccine status and ignorance of the presence of COVID-19 in Cameroon, as well as ignorance of the availability of vaccines, vaccination centers, and the cost of the vaccines. These associations were strengthened by logistic regression, which indicated that participants who were not aware of vaccination points were about five times more likely to not be vaccinated compared to others (add ratio: 5.18). Moreover, none of the participants who were unaware that the vaccines are available in Cameroon and are free of charge were vaccinated. Data collection for this study occurred in late April and March 2022 during the first vaccination campaign. A total of 840 vaccination centers were deployed, and a vast sensitization campaign was held with the objective of providing adequate information to at least 90% of the population [7,8]. Our results could signify that these targets were not met or that participants deliberately ignored vaccination centers in order to mark their vaccine hesitancy.

The lack of confidence towards COVID-19 vaccines was expressed by a significant association between beliefs about vaccine quality and deadly side effects, and, as a result, a preference for medicinal plants as a treatment option. In accordance with the conspiracy theory on the agenda of limiting the African population [20,25,28], 197 (37.88%) participants claimed that the vaccines were of bad quality, and 190 (36.54%) named potential severe side effects of vaccines, including gynecological cancer, sterility and death. None of the participants using the medicinal plants were vaccinated. The mistrust of vaccine quality has been reported as a leading factor towards vaccine hesitancy in many studies [29]. Our findings are also in accordance with the ideas that a high vaccine hesitancy rate is observed in people using medicinal plants or complementary medicines [12,30,31]. Considering the attitude towards COVID-19 vaccination, many researchers have focused on the curative potential of some medicinal plants against COVID-19 infection, and promising results have been revealed [32].

In our study, age and education level were also found to be significantly associated with vaccine acceptance. As older persons are more likely to be victims of COVID-19 complications, younger participants were less interested in the vaccines [33]. In contrast to those participants with primary and university education level, those with secondary education level were proportionally less vaccinated. Participants with a low level of education could be less exposed to social media and, thus, to misinformation, but this hypothesis was not verified in this study. These findings contrast with a study in France where participants with the lowest education level were more likely to refuse vaccines than other groups [34]. This study did not investigate the profession or the specific health personnel regarding their vaccination status, but many studies have reported disparities in vaccine acceptance among health personnel in Cameroon and abroad [6,35,36]. The investigation of this parameter in association with a patient’s decision to get vaccinated could help to provide in-depth information on the role of health personnel in vaccination coverage.

## 5. Conclusions

This study revealed that in the Menoua division, regardless of the effort of Cameroonian sanitary authorities, the vaccination rate is 10%. The lack of appropriate information, a fear of vaccine side effects, and sociodemographic features were identified as factors that have limited COVID-19 vaccine acceptance. These results indicate that the response strategy of Cameroon to disseminate COVID-19 vaccines should be reviewed, and that it should focus on sensitization in order to tackle the lack of information and/or misinformation among target groups. Local and effective solutions, including medicinal plants, could also be standardized and promoted.

## Figures and Tables

**Table 1:** Sociodemographic characteristics of the study population.

Sociodemographic Characteristics	Number	Frequency
Participants per subdivision		
Dschang	177	34.04
Fokoué	17	3.27
Fongo-Tongo	32	6.15
Nkong-Ni	106	20.38
Penka-Michel	128	24.62
Santchou	60	11.54
Gender		
Male	315	60.58
Female	205	39.42
Marital status		
Single	235	45.19
Married	285	54.81
Age groups		
<30	260	50.00
[30–50]	202	38.85
>50	58	11.15
Education level		
Primary	89	17.12
Secondary	292	56.15
University	139	26.73
Professions		
Civil servants	6	1.15
Informal sector	177	34.04
Health sector	21	4.04
Traders	177	34.04
Education sector	139	26.73

**Table 2:** Knowledge of the participants of COVID-19 manifestations, and its association with gender, education, and age.

Variables	Knowledge Level (*n* (%))			Total	*p* Value
	Good	Average	Null		
Gender					
Female	152 (74.15)	35 (17.07)	18 (8.78)	205	0.003
Male	189 (60.00)	88 (27.94)	38 (12.06)	315	
Total	341 [65.58]	123 [23.65]	56 [10.77]	520	
Education Level					
Primary	44 (49.44)	30 (33.70)	15 (16.85)	89	0.000
Secondary	184 (63.01)	73 (25.00)	35 (11.99)	292	
University	113 (81.29)	20 (14.39)	6 (4.32)	139	
Total	341 [65.58]	123 [23.65]	56 [10.77]	520	
Age Groups					
<30	174 (66.92)	60 (23.08)	26 (10.00)	260	0.178
[30–50]	131 (64.85)	50 (24.75)	21 (10.40)	202	
>50	36 (62.07)	13 (22.41)	9 (15.52)	58	
Total	341 [65.58]	123 [23.65]	56 [10.77]	520	

(): percentage in raw; []: percentage in the column.

**Table 3:** Knowledge of the participants on COVID-19 prevention and preventive methods.

Variables		Number	Frequency (%)
Knowledge of COVID-19 prevention			
Good		108	20.77
Average		376	72.31
Null		36	6.92
Total		520	100
Preventive methods			
Barriers measures		476	61.81
Medicinal plants		266	34.54
Total		770	100
Most cited plants			
Common names	Scientific names		
Ginger	*Zingiber officinale*	100	27.62
Lemon	*Citrus limon*	69	19.06
Garlic	*Allium sativum*	64	17.67
Artemisia	*Artemisia* sp.	62	17.12
Lemongrass	*Cymbopogon citratus*	27	7.47
Aloe vera	*Aloe vera*	26	7.19
Kinkeliba	*Combretum micranthum*	14	3.87

**Table 4:** Profile of medicinal plants users as a preventive method against COVID-19.

Variables	Gender		Age Groups			Education		
Modality	Female	Male	<30	[30–50]	>50	Primary	Secondary	University
Number	108	158	120	113	33	49	137	80
%	52.68	50.16	46.15	55.94	56.90	55.06	46.92	57.55
*p*-value	0.64		0.004			0.08		

%: Percentage of the total modality in the sample.

**Table 5:** Knowledge and attitude of the participants towards the COVID-19 vaccine.

Variables	Modalities	Number	Frequency (%)
Availability of the vaccine in Cameroon	Don’t know	22	4.23
	Not available	62	11.92
	Available	436	83.85
Nearest vaccination center	Don’t know	216	41.54
	Know	304	58.56
Role of vaccines	Prevent hospitalization	40	7.69
	Prevent contamination	379	92.31
Quality of vaccines	Don’t know	180	34.61
	Good quality	143	27.5
	Bad quality	197	45.18
Side effects	Gynaecologic cancers	21	4.04
	Sterility	32	6.15
	Death	137	26.35
	Other	330	63.46
Worried about side effects	No	218	41.92
	A bit	67	12.88
	Very	235	45.19

**Table 6:** Association between knowledge and COVID-19 vaccine status in the Menoua Division.

Variables	Vaccinal Status				*p*-Value	Odd Ratio
	Vaccinated		Non Vaccinated			
	Number	Frequency	Number	Frequency		
Presence of COVID-19 in Cameroon						
No	6	6.66	84	93.34	0.85	1.67
Yes	46	10.70	384	89.30		
Total	52	10.00	468	90.00		
Availability of vaccines in Cameroon						
No	0	0.00	84	100.00	0.001	1.135
Yes	52	11.92	384	88.08		
Total	52	10.00	468	90.00		
Knowledge on the nearest vaccination center						
Don’t know	7	3.24	209	96.76	0.000	5.18
Know	45	14.80	259	85.20		
Total	52	52.00	468	90.00		
Are vaccines free						
Yes	52	17.85	451	82.15	0.000	17.58
No	0	0.00	17	100.00		
Total	52	10.00	468	90.00		

**Table 7:** Association between the population’s attitude and COVID-19 vaccine status in the Menoua Division.

Variables	Vaccination Status				*p*-Value
	Vaccinated		Non-Vaccinated		
	Number	%	Number	%	
Vaccine quality					
Good	43	82.69	100	21.37	0.000
Bad	4	7.69	193	41.24	
Don’t know	5	9.62	175	37.39	
Total	52	100.00	468	100.00	
Side effects					
Gynaecologic cancer	2	3.85	19	4.06	0.002
Sterility	3	5.77	29	6.20	
Death	3	5.77	134	28.63	
Others	44	84.62	286	61.11	
Total	52	100.00	468	100.00	
Worried about side effects					
No	38	73.08	180	38.46	0.000
A bit	5	9.62	62	13.25	
Very	9	17.31	226	48.29	
Total	52	100.00	468	100.00	
Plants provide better prevention					
Yes	0	0.00	88	100.00	0.000
No	52	10.00	380	90.00	
Total	52	10.00	468	90.00	

**Table 8:** Sociodemographic factors associated with COVID-19 vaccine status in the Menoua Division.

Variables		Vaccination Status			*p*-Value
	Vaccinated		Non-Vaccinated		
	Number	Frequency	Number	Frequency	
Age groups					
<30	18	34.62	242	51.71	0.00
[30–50]	24	46.15	178	38.03	
>50	10	19.23	48	10.26	
Total	52	100.00	468	100.00	
Gender					
Female	24	46.15	181	38.68	0.184
Male	28	53.85	287	61.32	
Total	52	100.00	468	100.00	
Education level					
Primary	11	21.15	78	16.67	0.019
Secondary	20	38.46	272	58.12	
University	21	40.38	118	25.21	
Total	52	100.00	468	100.00	

## Data Availability

The datasets used and/or analyzed during the current study are available from the corresponding author on reasonable request.
